# Supplementary material for: Scattering approach to diffusion quantifies axonal damage in brain injury
Source: ArXiv. 2025 Sep 16:arXiv:2501.18167v2. Originally published 2025 Jan 30. Preprint. [Version 2] (PMC11838777)
Supplement: Supplement 1 [file NIHPP2501.18167v2-supplement-1.pdf]

Supplementary Information

Scattering approach to diffusion quantifies axonal damage in brain injury

Ali Abdollahzadeh[1,2,*], Ricardo Coronado-Leija[1], Hong-Hsi Lee[3], Alejandra Sierra[2], Els Fieremans[1], Dmitry S. Novikov[1,*]

[1]Center for Biomedical Imaging, Department of Radiology, New York University School of Medicine, New York, NY, USA
[2]A.I. Virtanen Institute for Molecular Sciences, University of Eastern Finland, Kuopio, Finland
[3]Athinoula A. Martinos Center for Biomedical Imaging, Department of Radiology, Massachusetts General Hospital, Harvard Medical School, Boston, MA, USA
[*]ali.abdollahzadeh@uef.fi
[*]dmitry.novikov@nyulangone.org

ESTIMATION OF THE $\Gamma_\eta(q)$ LIMIT Γ_0 AT $q \to 0$

Determining the $q \to 0$ limit Γ_0 from the power spectral density $\Gamma_\eta(q)$ of axons, especially for shorter axons with only a few low-q Fourier harmonics, requires a robust solution, Fig. S1. For that we practically fit a polynomial of the form $\gamma q^2 + \Gamma_0$ to $\Gamma_\eta(q)$ in the range $[q_{\min},\ q_{\max}]$, where $q_{\min} = 2\pi/L$ is the smallest accessible q, and $q_{\max}$ is defined as the spatial frequency for which the small-q variance of the fluctuations is the fraction β of the total variance, i.e.,

$$\int_0^{q_{\max}} \frac{dq}{2\pi}\, \Gamma_\eta(q) = \beta \cdot \int_0^{\infty} \frac{dq}{2\pi}\, \Gamma_\eta(q). \qquad \text{(S1)}$$

We empirically fixed $\beta = 0.93$ for all EM axons and 0.98 for all synthetic axons in our analysis, Fig. S1.

Coarse-graining over the increasing diffusion length $\ell(t)$ suppresses $\Gamma_\eta(q)$, such that only the limit (the "plateau") Γ_0 survives for long t and governs the diffusive dynamics. Our approach to estimating Γ_0 as the constant term in the polynomial fit $\gamma q^2 + \Gamma_0$, is consistent with this physical picture. In Fig. 2d of the main text, the estimated $\Gamma_0 = 0.21 \pm 0.03$ (mean±std) remained unchanged for a range of diffusion times $t_0, \ldots, t_3$. The magnitude of the quadratic term γ increased substantially during coarse-graining: $\gamma = -5.23, -123.61, -265.04, -1331$ for $t_0, \ldots, t_3$, indicating a progressively stronger suppression of the finite-q Fourier harmonics.

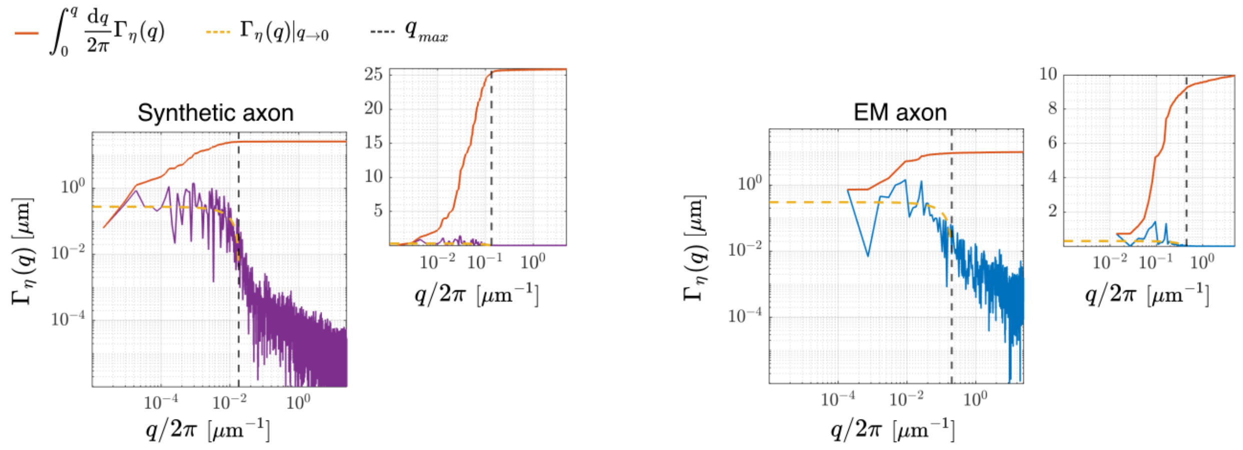

FIG. S1. **Estimation of Γ_0.** Estimating Γ_0 from $\Gamma_\eta(q)$ for short axons with only a few low-q Fourier harmonics can be noisy. The $L = 500\,\mu$m synthetic axons (left panel) provide access to notably more relevant points at $q \to 0$ compared to the $L = 73.6\,\mu$m EM axons (right panel) for defining the plateau Γ_0, with the polynomial fit shown by the yellow dashed line. The vertical dashed line indicates $q_{\max}$ determined via Eq. (S1). Smaller insets show the same plots with the Γ_η-axis in the normal, rather than log scale.

EFFECT OF CHRONIC TBI ON AXON MORPHOLOGY AND $D(t)$

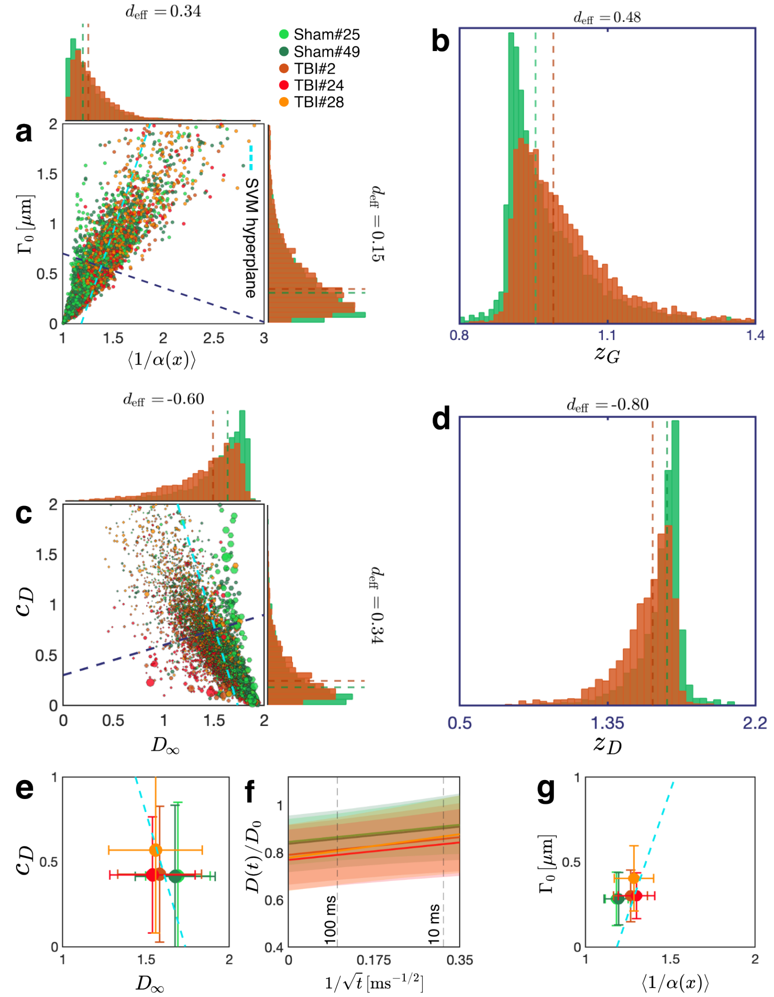

FIG. S2. **Effect of chronic TBI on axon morphology and D(t) from the contralateral cingulum.** **(a)** Geometric tortuosity $\langle 1/\alpha \rangle$, Eq. (2), and the variance Γ_0 of long-range cross-sectional fluctuations entering Eq. (3), are plotted for myelinated axons segmented from the contralateral cingulum of sham-operated (shades of green; $N_{\text{axon}} = 4,580$) and TBI (shades of red; $N_{\text{axon}} = 4,580$) rats. **(b)** The optimal linear combination z_G of the morphological parameters is derived from a trained support vector machine (SVM). Projecting the points onto the dark blue dashed line in **(a)** perpendicular to the SVM hyperplane constitutes the maximal separation between the two groups. **(c)** Predicted individual axon diffusion parameters $D_{\infty,i}$ and $c_{D,i}$ from Eqs. (2)–(3) plotted for myelinated axons in **(a)**. The size of each point reflects its weight w_i in the net dMRI-accessible $D(t)$, proportional to the axon volume. **(d)** The optimal SVM-based linear combination z_D of the diffusion parameters is derived by projecting the points onto the dark blue dashed line in **(c)** perpendicular to the corresponding SVM hyperplane. Dashed lines in **(a-d)** indicate the medians of the distributions. **(e)** The macroscopic diffusivity parameters c_D and D_∞ for each animal are obtained by volume-weighting (filled circles; $N = 2$ sham-operated and $N = 3$ TBI) the individual axonal contributions $D_{\infty,i}$ and $c_{D,i}$. Error bars represent measurement uncertainties in the volume-weighted estimates (see *Methods*). The SVM hyperplane (cyan dashed line) is the same as that for the diffusion parameters of individual axons in **(c)**. **(f)** Predicting the along-tract $D(t)/D_0$ as a function of $1/\sqrt{t}$, Eq. (1), based on the overall D_∞ and c_D in **(e)**. **(g)** The effect of TBI on the ensemble-averaged geometry (filled circles) is illustrated by transforming the macroscopic ensemble diffusivity in **(e,f)**, as if from an MRI measurement, back onto the space of morphological parameters $\langle 1/\alpha \rangle$ and Γ_0, via inverting Eqs. (2)–(3). The SVM hyperplane (cyan dashed line) is the same as that for the morphological parameters of individual axons in **(a)**. Error bars corresponding to standard deviations of $D(t)/D_0$ in **(f)** and $\langle 1/\alpha \rangle$ and Γ_0 in **(g)** are calculated based on errors in **(e)** (see *Methods*). Source data are provided as a Source Data file.

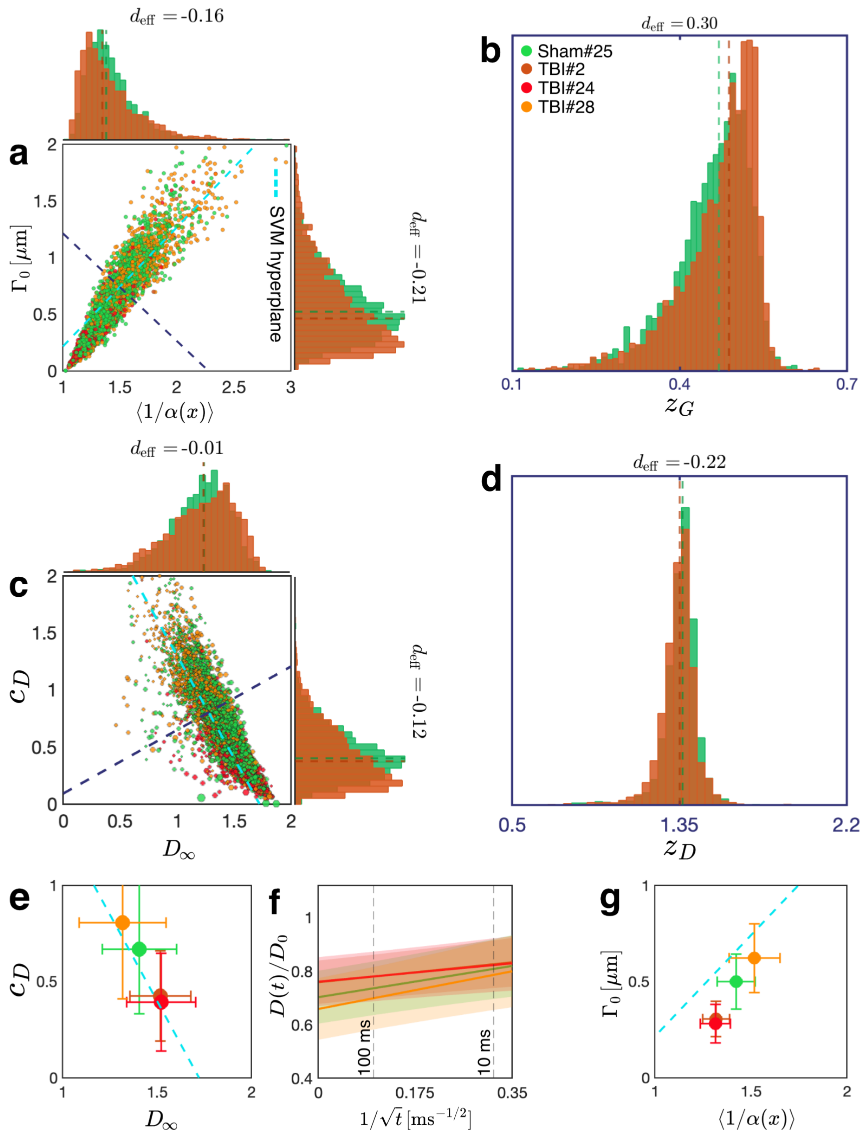

FIG. S3. **Effect of chronic TBI on axon morphology and D(t) from the ipsilateral corpus callosum.** **(a)** Geometric tortuosity $\langle 1/\alpha\rangle$, Eq. (2), and the variance Γ_0 of long-range cross-sectional fluctuations entering Eq. (3), are plotted for myelinated axons segmented from the ipsilateral corpus callosum of sham-operated (shades of green; $N_{\text{axon}} = 2,703$) and TBI (shades of red; $N_{\text{axon}} = 2,703$) rats. **(b)** The optimal linear combination z_G of the morphological parameters is derived from a trained support vector machine (SVM). Projecting the points onto the dark blue dashed line in **(a)** perpendicular to the SVM hyperplane constitutes the maximal separation between the two groups. **(c)** Predicted individual axon diffusion parameters $D_{\infty,i}$ and $c_{D,i}$ from Eqs. (2)–(3) plotted for myelinated axons in **(a)**. The size of each point reflects its weight w_i in the net dMRI-accessible $D(t)$, proportional to the axon volume. **(d)** The optimal SVM-based linear combination z_D of the diffusion parameters is derived by projecting the points onto the dark blue dashed line in **(c)** perpendicular to the corresponding SVM hyperplane. Dashed lines in **(a-d)** indicate the medians of the distributions. **(e)** The macroscopic diffusivity parameters c_D and D_∞ for each animal are obtained by volume-weighting (filled circles; $N = 1$ sham-operated and $N = 3$ TBI) the individual axonal contributions $D_{\infty,i}$ and $c_{D,i}$. Error bars represent measurement uncertainties in the volume-weighted estimates (see *Methods*). The SVM hyperplane (cyan dashed line) is the same as that for the diffusion parameters of individual axons in **(c)**. **(f)** Predicting the along-tract $D(t)/D_0$ as a function of $1/\sqrt{t}$, Eq. (1), based on the overall D_∞ and c_D in **(e)**. **(g)** The effect of TBI on the ensemble-averaged geometry (filled circles) is illustrated by transforming the macroscopic ensemble diffusivity in **(e,f)**, as if from an MRI measurement, back onto the space of morphological parameters $\langle 1/\alpha\rangle$ and Γ_0, via inverting Eqs. (2)–(3). The SVM hyperplane (cyan dashed line) is the same as that for the morphological parameters of individual axons in **(a)**. Error bars corresponding to standard deviations of $D(t)/D_0$ in **(f)** and $\langle 1/\alpha\rangle$ and Γ_0 in **(g)** are calculated based on errors in **(e)** (see *Methods*). Source data are provided as a Source Data file.

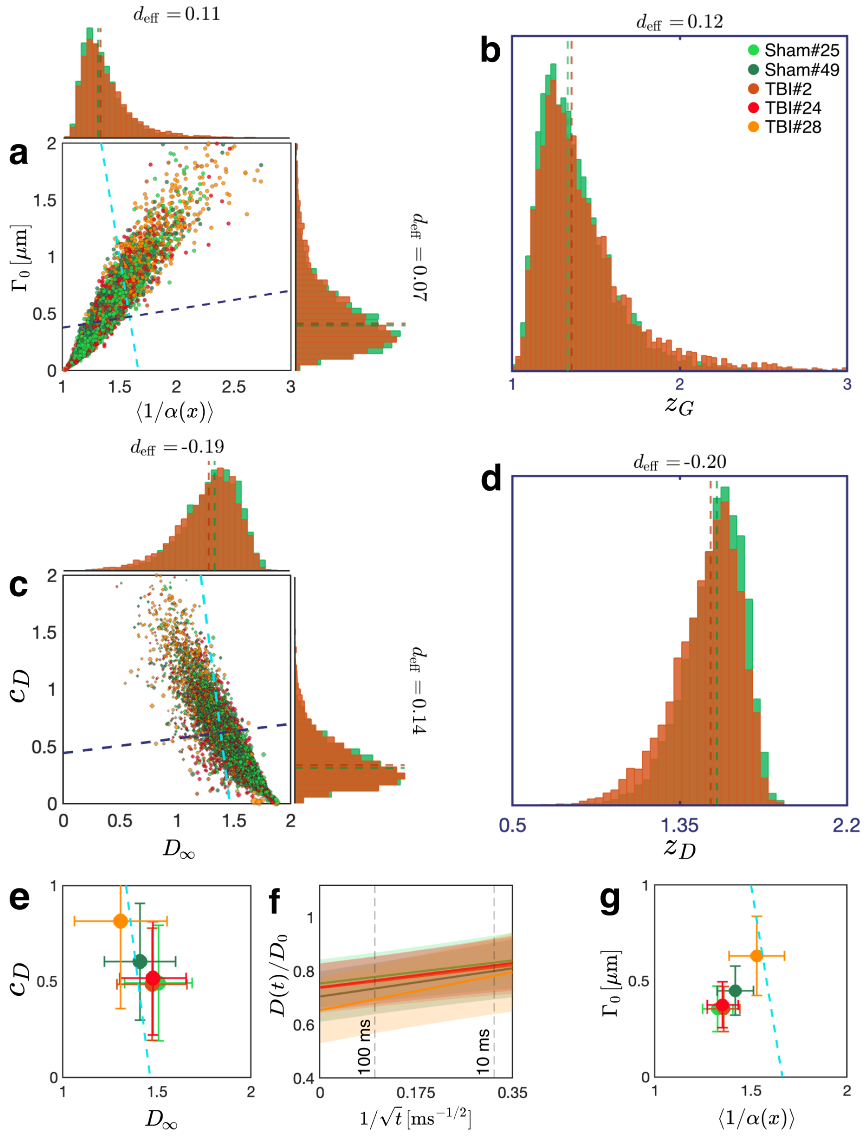

FIG. S4. **Effect of chronic TBI on axon morphology and D(t) from the contralateral corpus callosum.** **(a)** Geometric tortuosity $\langle 1/\alpha \rangle$, Eq. (2), and the variance Γ_0 of long-range cross-sectional fluctuations entering Eq. (3), are plotted for myelinated axons segmented from the contralateral corpus callosum of sham-operated (shades of green; $N_{\text{axon}} = 4,080$) and TBI (shades of red; $N_{\text{axon}} = 4,080$) rats. **(b)** The optimal linear combination z_G of the morphological parameters is derived from a trained support vector machine (SVM). Projecting the points onto the dark blue dashed line in **(a)** perpendicular to the SVM hyperplane constitutes the maximal separation between the two groups. **(c)** Predicted individual axon diffusion parameters $D_{\infty,i}$ and $c_{D,i}$ from Eqs. (2)–(3) plotted for myelinated axons in **(a)**. The size of each point reflects its weight w_i in the net dMRI-accessible $D(t)$, proportional to the axon volume. **(d)** The optimal SVM-based linear combination z_D of the diffusion parameters is derived by projecting the points onto the dark blue dashed line in **(c)** perpendicular to the corresponding SVM hyperplane. Dashed lines in **(a-d)** indicate the medians of the distributions. **(e)** The macroscopic diffusivity parameters c_D and D_∞ for each animal are obtained by volume-weighting (filled circles; $N = 2$ sham-operated and $N = 3$ TBI) the individual axonal contributions $D_{\infty,i}$ and $c_{D,i}$. Error bars represent measurement uncertainties in the volume-weighted estimates (see *Methods*). The SVM hyperplane (cyan dashed line) is the same as that for the diffusion parameters of individual axons in **(c)**. **(f)** Predicting the along-tract $D(t)/D_0$ as a function of $1/\sqrt{t}$, Eq. (1), based on the overall D_∞ and c_D in **(e)**. **(g)** The effect of TBI on the ensemble-averaged geometry (filled circles) is illustrated by transforming the macroscopic ensemble diffusivity in **(e,f)**, as if from an MRI measurement, back onto the space of morphological parameters $\langle 1/\alpha \rangle$ and Γ_0, via inverting Eqs. (2)–(3). The SVM hyperplane (cyan dashed line) is the same as that for the morphological parameters of individual axons in **(a)**. Error bars corresponding to standard deviations of $D(t)/D_0$ in **(f)** and $\langle 1/\alpha \rangle$ and Γ_0 in **(g)** are calculated based on errors in **(e)** (see *Methods*). Source data are provided as a Source Data file.

EFFECT OF MILD TBI ON TIME-DEPENDENT AXIAL $D(t)$ AND ON AXON MORPHOLOGY FROM *EX VIVO* DTI

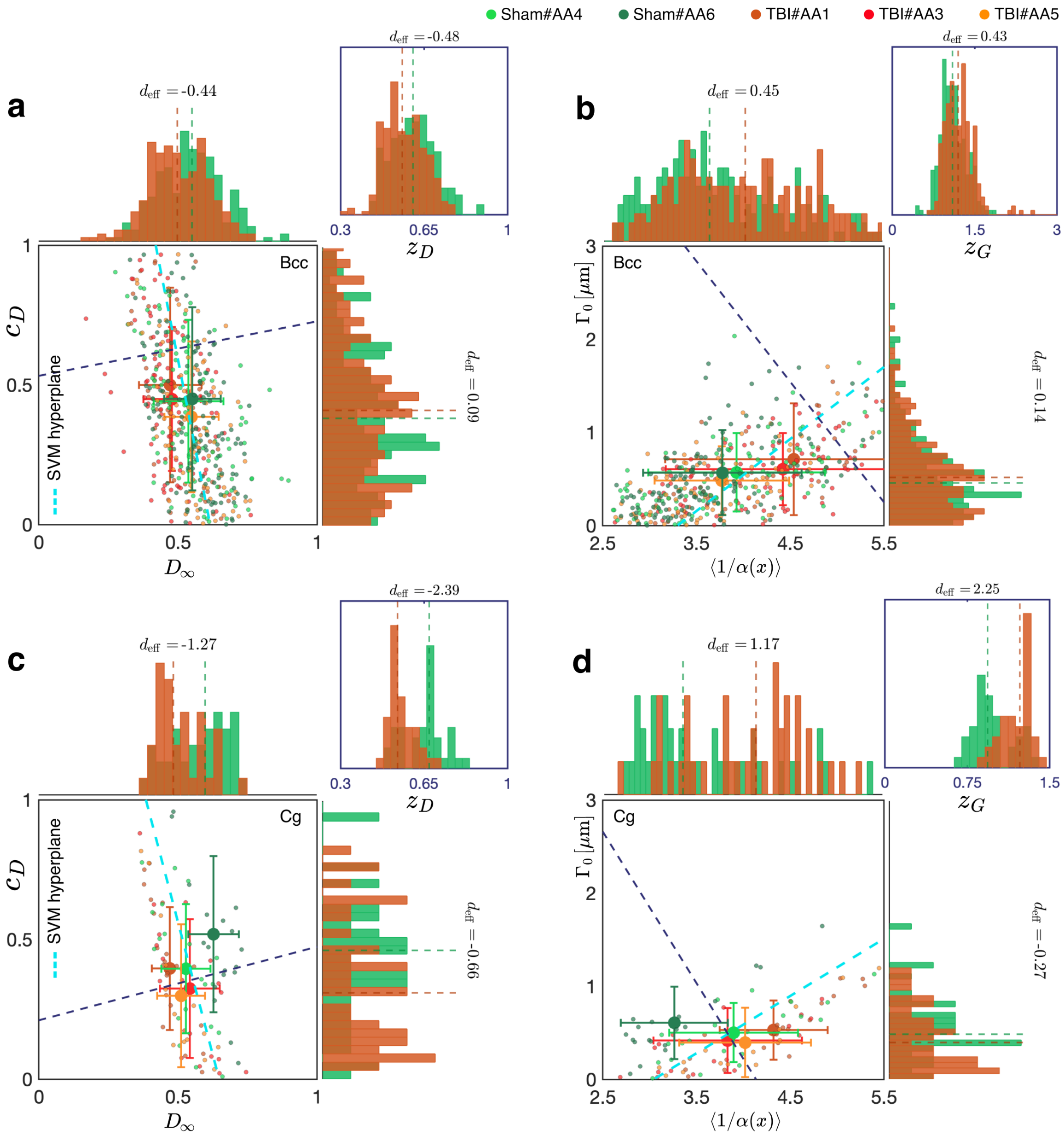

FIG. S5. **Effect of mild TBI on *ex vivo* dMRI and axon morphology in ipsilateral Bcc and Cg.** **(a) and (c)** Diffusion parameters D_∞ and c_D extracted by linear regression of $D(t)$ with respect to $1/\sqrt{t}$ for voxels within the ipsilateral Bcc ($N_{\text{voxel}} = 240$ per group) and Cg ($N_{\text{voxel}} = 39$ per group) ROIs. The optimal SVM-based linear combination z_D of the diffusion parameters is derived by projecting the points onto the dark blue dashed line perpendicular to the corresponding SVM hyperplane. **(b) and (d)** Corresponding geometric parameters $\langle 1/\alpha \rangle$ and Γ_0, computed by inverting Eqs. (2)–(3) from the diffusion parameters, plotted for voxels in Bcc and Cg. The optimal linear combination z_G of the morphological parameters is derived by projecting the points onto the dark blue dashed line perpendicular to the SVM hyperplane. In all panels, each point represents a voxel. Filled circles with error bars indicate the mean and standard deviation across the ROI ($N = 2$ sham-operated and $N = 3$ TBI). Dashed vertical lines overlaid on the distributions denote the medians. Source data are provided in the Source Data file.

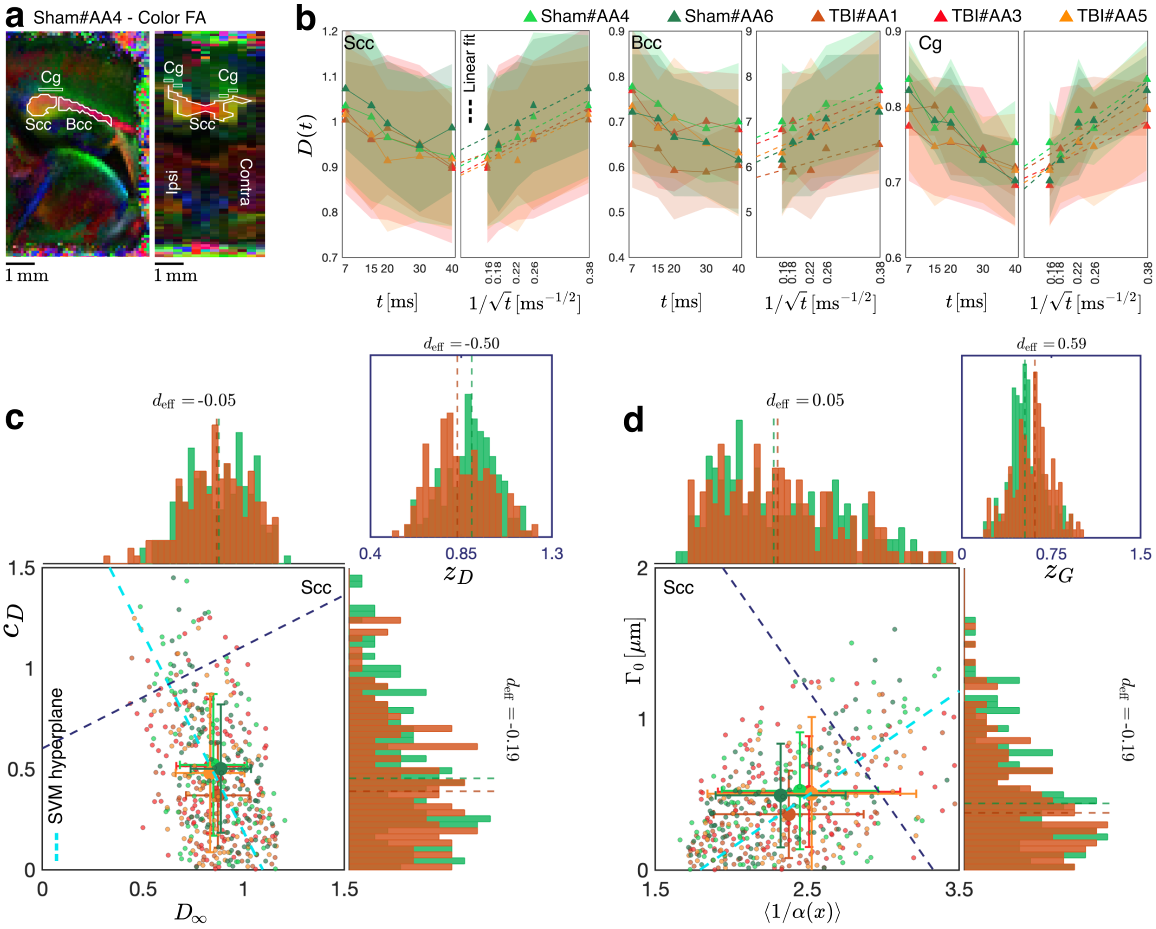

FIG. S6. **Effect of mild TBI on *ex vivo* dMRI and axon morphology in contralateral Scc.** **(a)** Representative colored fractional anisotropy (FA) maps in sagittal and coronal views, with the cingulum (Cg), splenium of the corpus callosum (Scc), and body of the corpus callosum (Bcc) annotated. **(b)** Experimental axial DTI diffusivity $D(t)$ plotted as a function of t and $1/\sqrt{t}$, showing a power-law relation in all contralateral white matter regions of interest (ROIs). **(c)** Diffusion parameters D_∞ and c_D extracted by linear regression of $D(t)$ with respect to $1/\sqrt{t}$ in **(b)** for voxels within the contralateral Scc ROI ($N_{\rm voxel} = 186$ per group). The optimal SVM-based linear combination z_D of the diffusion parameters is derived by projecting the points onto the dark blue dashed line perpendicular to the corresponding SVM hyperplane. **(d)** Corresponding geometric parameters $\langle 1/\alpha \rangle$ and Γ_0, computed by inverting Eqs. (2)–(3) from the diffusion parameters in **(c)**, plotted for voxels in Scc. The optimal linear combination z_G of the morphological parameters is obtained by projecting the data points onto the dark blue dashed line, which is orthogonal to the SVM hyperplane. In **(b)**, filled triangles with shaded areas indicate the mean and standard deviation across the ROI ($N = 2$ sham-operated and $N = 3$ TBI). In **(c)**–**(d)**, each point represents a voxel. Filled circles with error bars indicate the mean and standard deviation across the ROI. Dashed vertical lines overlaid on the distributions denote the medians. Source data are provided in the Source Data file.

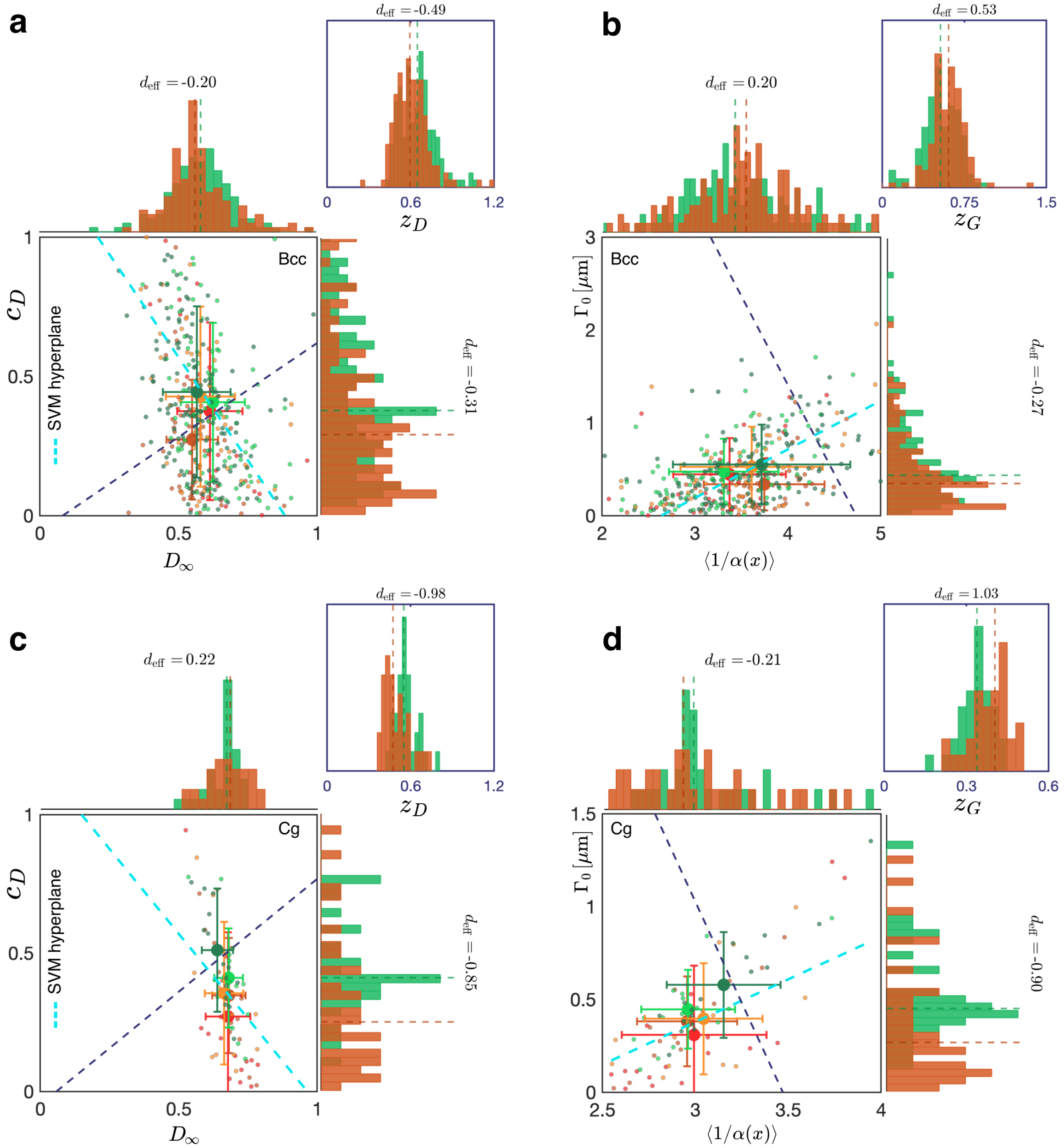

FIG. S7. **Effect of mild TBI on *ex vivo* dMRI and axon morphology in contralateral Bcc and Cg. (a) and (c)** Diffusion parameters D_∞ and c_D extracted by linear regression of $D(t)$ with respect to $1/\sqrt{t}$ for voxels within the contralateral Bcc ($N_{\rm voxel} = 148$ per group) and Cg ($N_{\rm voxel} = 33$ per group) ROIs. The optimal SVM-based linear combination z_D of the diffusion parameters is derived by projecting the points onto the dark blue dashed line perpendicular to the corresponding SVM hyperplane. **(b) and (d)** Corresponding geometric parameters $\langle 1/\alpha \rangle$ and Γ_0, computed by inverting Eqs. (2)–(3) from the diffusion parameters, plotted for voxels in Bcc and Cg. The optimal linear combination z_G of the morphological parameters is derived by projecting the points onto the dark blue dashed line perpendicular to the SVM hyperplane. In all panels, each point represents a voxel. Filled circles with error bars indicate the mean and standard deviation across the ROI ($N = 2$ sham-operated and $N = 3$ TBI). Dashed vertical lines overlaid on the distributions denote the medians. Source data are provided in the Source Data file.

MOMENTS OF AXON RADIUS DISTRIBUTION CONTRIBUTING TO THE TORTUOSITY VERSUS EFFECTIVE AXON RADIUS

For a heterogeneous axon population, both the tortuosity $\langle 1/\alpha \rangle$ and the effective MR radius [124] $r_{\rm eff}$ in previously developed axon radius mapping with dMRI [125] (by applying extremely strong gradients) involve the moments of axon radius distribution. Here, we compare the contributions of such moments to both quantities and show that tortuosity provides a similar degree of separation between TBI and sham rats, yet it involves lower-order moments of radius.

Effective axon MR radius for an ensemble of irregularly-shaped axons

Diffusion MRI-measured effective axon radius (in the wide-pulse regime) corresponds to the ratio of the 6th and 2nd moments of the distribution of straight cylinders [124]:

$$r_{\rm eff} = \left(\frac{\langle r^6 \rangle_{\rm ensemble}}{\langle r^2 \rangle_{\rm ensemble}} \right)^{1/4} . \tag{S2}$$

This comes from the Neuman's result [126] for the signal attenuation, $-\ln S \propto r^4$, that is subsequently volume-weighted, such that effectively, $r_{\rm eff}^4$ is measured to the lowest order in the diffusion attenuation over the ensemble of cylinders.

Recently, it was shown [59] that the same expression (S2) applies for an effective MR radius measured in a *single axon* with variable cross-section, i.e., the cross-sections of an irregularly-shaped axon in 3 dimensions effectively act as an ensemble of independent 2-dimensional disks (or uniform cylinders). In other words, for a single axon, the effective MR radius is given by Eq. (S2) where the ensemble averaging $\langle \dots \rangle_{\rm ensemble} \to \langle \dots \rangle = \frac{1}{L} \int \mathrm{d}x \dots$ is substituted by the average along the axon axis x, as in the main text.

In our case of an ensemble of irregularly shaped axons, the averaging both along each axon axis and over the ensemble of axons yields

$$r_{\rm eff}^4 = \sum_i w_i \, r_{\rm eff,i}^4 = \frac{\sum_i \langle r_i^2(x) \rangle \langle r_i^6(x) \rangle / \langle r_i^2(x) \rangle}{\sum_j \langle r_j^2(x) \rangle} = \frac{\sum_i \langle r_i^6(x) \rangle}{\sum_j \langle r_j^2(x) \rangle} \tag{S3}$$

where, as discussed in the main text, the weights are proportional to the mean cross-sectional area $\bar{A}_i = \langle A_i(x) \rangle$,

$$w_i = \frac{\bar{A}_i}{\sum_j \bar{A}_j} = \frac{\langle r_i^2(x) \rangle}{\sum_j \langle r_j^2(x) \rangle} , \quad \text{where} \quad A_i(x) = \pi r_i^2(x) \tag{S4}$$

defines the equivalent radius $r_i(x)$ (as always, we assume that axons have the same length). As everywhere in this paper, the angular brackets denote averaging along the axon axis x. In other words, the effective radius (S3) corresponds to the ratio of the 6th and 2nd moments of the *joint distribution of equivalent axon radii* over cross-sections and axons — equivalent to slicing all axons into individual cross-sections and pooling all of them into one distribution.

Effective tortuosity for an ensemble of irregularly-shaped axons

Consider now the ensemble-averaged D_∞, assuming the same D_0 for all axons:

$$\frac{D_{\infty,\rm eff}}{D_0} = \sum_i w_i \frac{D_{\infty,i}}{D_0} = \sum_i \frac{w_i}{\langle 1/\alpha_i(x) \rangle} = \sum_i \frac{w_i}{\langle 1/(1+\delta\alpha_i(x)) \rangle} , \tag{S5}$$

where expanding $\langle 1/(1+\delta\alpha(x)) \rangle$ up to $(\delta\alpha(x))^2$, we can write Eq. (S5) as

$$\frac{D_{\infty,\rm eff}}{D_0} = \sum_i \frac{w_i}{1 + \langle (\delta\alpha_i(x))^2 \rangle + \mathcal{O}\left((\delta\alpha_i(x))^3 \right)} \simeq \sum_i w_i \left(1 - \langle (\delta\alpha_i(x))^2 \rangle \right) . \tag{S6}$$

Using the weights (S4) and $\langle(\delta\alpha_i(x))^2\rangle = \langle A_i^2(x)\rangle/\bar{A}_i^2 - 1$, we can rewrite Eq. (S6) via the equivalent radii $r_i(x)$ as

$$\frac{D_{\infty,\mathrm{eff}}}{D_0} \simeq 1 - \left[\frac{1}{\sum_j \langle r_j^2(x)\rangle} \sum_i \frac{\langle r_i^4(x)\rangle}{\langle r_i^2(x)\rangle} - 1\right] \quad \Rightarrow \quad \frac{D_0}{D_{\infty,\mathrm{eff}}} \simeq \frac{1}{\sum_j \langle r_j^2(x)\rangle} \sum_i \frac{\langle r_i^4(x)\rangle}{\langle r_i^2(x)\rangle}\,. \tag{S7}$$

Thus, the tortuosity is dominated by the 2nd and 4th moments of equivalent radii (i.e., less affected by the tail of the radius distribution). Yet, it cannot be expressed via moments of the *joint distribution* over cross-sections and axons.

Separating sham-operated and TBI with ensemble-averaged r_{eff} and tortuosity

In Fig. S8, we compare the sensitivity of the effective tortuosity Eq. (S5) with that of the effective radius r_{eff} Eq. (S3). Both measures have approximately similar sensitivity in separating sham-operated and TBI datasets for all comparisons.

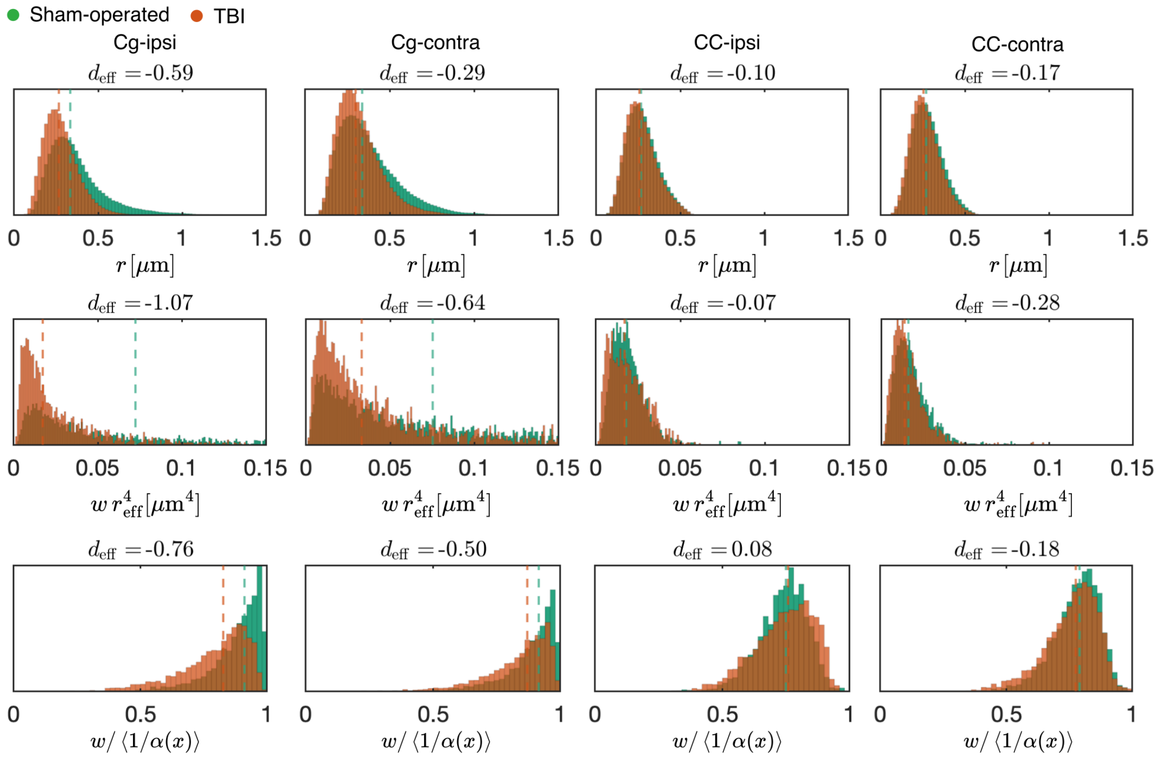

FIG. S8. **Sensitivity of tortuosity versus effective radius**. The top row shows histograms of axon radius across all axons and cross-sections in each considered brain region: Cg-ipsi ($N_{\mathrm{axon}} = 3,999$), Cg-contra ($N_{\mathrm{axon}} = 4,580$), CC-ipsi ($N_{\mathrm{axon}} = 2,703$), and CC-contra ($N_{\mathrm{axon}} = 4,080$). The histograms demonstrate the decrease in the axon radius caused by TBI on the ipsilateral side. The middle row shows histograms of volume-weighted effective MR radius wr_{eff}^4 across all axons in each region, entering the weighted average in Eq. (S3). The bottom row shows histograms of volume-weighted reciprocal tortuosity $w/\langle 1/\alpha(x)\rangle$ across all axons in each region, entering the weighted average in Eq. (S5). The reciprocal tortuosity (effectively averaged in a bulk measurement of D_∞) and the 4th power of effective MR radius r_{eff} (effectively averaged in a bulk measurement of axon radius) show similar effect sizes for separating sham-operated and TBI datasets. However, estimating r_{eff} requires very strong diffusion gradients. Dashed lines are the medians of the underlying distributions, exemplifying the macroscopically measured quantities. Source data are provided in the Source Data file.

THE SINGLE-BEAD MODEL

To get an intuition for the key quantity Γ_0 determining the amplitude Eq. (3) of the $1/\sqrt{t}$ tail Eq. (1) in $D(t)$, here we consider the *single-bead model* of a randomly-shaped axon. Namely, as mentioned in the main text, we represent an axon of length L with a normalized cross-sectional area $\alpha(x) = A(x)/\bar{A} \equiv e^{\eta(x)}$ as a set of N identical multiplicative beads with the shape $\eta_1(x)$ placed at random positions x_m on top of the uniform "background" η_0 without beads:

$$\eta(x) = \ln \alpha(x) = \eta_0 + \sum_{m=1}^{N} \eta_1(x - x_m)\,, \tag{S8}$$

such that the macroscopic bead density $\bar{n} = N/L$. For the power spectral density (19), we need the Fourier transform

$$\eta(q) = \eta_0 \cdot 2\pi\delta(q) + \eta_1(q) \sum_{m=1}^{N} e^{-iqx_m}\,. \tag{S9}$$

Writing the square of the Dirac delta-function $\delta^2(q) = \delta(q)\delta(q=0) = (L/2\pi)\delta(q)$ (assuming $L \to \infty$), we obtain

$$\Gamma_\eta(q) = \frac{|\eta(q)|^2}{L} = 2\pi\eta_0 \left[\eta_0 + 2\frac{\zeta}{\bar{a}}\right] \delta(q) + |\eta_1(q)|^2 \Gamma_{\rm pos}\,, \quad \Gamma_{\rm pos}(q) = \frac{1}{L} \sum_{m,m'=1}^{N} e^{-iq(x_m - x_{m'})}\,, \tag{S10}$$

where the "bead length"

$$\zeta = \eta_1(q)|_{q=0} = \int \mathrm{d}x\, \eta_1(x)\,, \tag{S11}$$

and $\bar{a} = L/N = 1/\bar{n}$ is the mean interval between successive bead positions (i.e., the inverse number density $\bar{n}$).

Equation (S10) is so far very general. The statistics of bead placement are reflected in the particular functional form of the power spectral density $\Gamma_{\rm pos}(q)$ of bead positions. In our model, we further assume that the successive intervals $a_m = x_{m+1} - x_m$ are independent and identically distributed random variables chosen from a probability density function (PDF) $P(a)$ with a finite mean $\bar{a}$ and variance σ_a. This allows us to represent $\Gamma_{\rm pos}(q)$ in terms of the parameters of $P(a)$. Assuming self-averaging in a large enough system ($N \gg 1$), we can substitute $\Gamma_{\rm pos}(q)$ for a particular disorder realization by its expected value

$$\Gamma_{\rm pos}(q) = \frac{1}{L} \sum_{m,m'=1}^{N} \left\langle e^{-iq(x_m - x_{m'})} \right\rangle = \frac{1}{L} \sum_{m=1}^{N} \left\langle 1 + \sum_{s=1} e^{-iq(a_1 + \cdots + a_s)} + \sum_{s=1} e^{iq(a_1 + \cdots + a_s)} \right\rangle = \bar{n} \left[1 + \frac{\tilde{p}_q}{1 - \tilde{p}_q} + \frac{\tilde{p}_q^*}{1 - \tilde{p}_q^*}\right], \tag{S12}$$

where $\tilde{p}_q = \int \mathrm{d}a\, e^{-iqa} P(a)$ is the characteristic function of $P(a)$, and $*$ stands for the complex conjugation. The above disorder averaging was performed by representing $x_m - x_{m'} = \sum_{j=m'}^{m-1} a_j$, splitting the double sum into three terms (with $m = m'$, $m > m'$, and $m < m'$, where $s = m - m'$), and summing the geometric series in the limit $N = \bar{n}L \to \infty$. Representing the characteristic function $\tilde{p}_q$ via its cumulants, $\tilde{p}_q = e^{-iq\bar{a} - q^2\sigma_a^2/2 + \cdots}$ in Eq. (S12) and taking the limit $q \to 0$ by expanding $\tilde{p}_q$ up to q^2 yields

$$\Gamma_{\rm pos}(q)|_{q\to 0} = \frac{\sigma_a^2}{\bar{a}^3}\,. \tag{S13}$$

Plugging Eq. (S13) into Eq. (S10) and taking the $q \to 0$ limit, we finally obtain [51, 54]

$$\Gamma_0 = \Gamma_\eta(q)|_{q\to 0} = \frac{\sigma_a^2}{\bar{a}} \phi^2\,, \quad \phi = \frac{\zeta}{\bar{a}}\,, \tag{S14}$$

where the factor $\sigma_a^2/\bar{a}$ characterizes the statistics of bead positions, and ϕ is a dimensionless length ratio that tells how pronounced the relative area modulation is.

In Fig. S9, we decompose Γ_0 into its comprising factors, according to Eq. (S14), and quantify their separate TBI effect sizes in different brain regions. Rather than modeling the explicit bead shape $\eta_1(x)$, we quantified its integrated "bead length" (S11) by integrating (within each interval a_m between the successive minima of $\eta(x)$) the excess $\eta(x) - \eta_0$ relative to the baseline η_0 defined as the global minimum of $\eta(x)$ for each axon, and averaging such integrals over all

intervals a_m for each axon to obtain ζ. To determine ϕ, we further used Eq. (S14), where $\bar{a}$ was estimated as the average distance between successive local minima of $\eta(x)$.

In Fig. S10, we analyze the bead position statistics and their change in TBI in greater detail. The top row of Fig. S10 shows that the mean distance between successive beads $\bar{a}$ decreases (and the bead density $\bar{n}$ increases) in TBI axons compared to sham-operated animals. Notably, bead positions in TBI axons exhibit a smaller coefficient of variation $\sigma_a/\bar{a}$, suggesting a more ordered arrangement of the beads (middle row). To explore this further, we considered the power spectral density $\Gamma_{\text{pos}}(q)$ of bead positions, Eq. (S10). The finite plateau $\bar{a} \cdot \Gamma_{\text{pos}}|_{q\to 0} = (\sigma_a/\bar{a})^2$ in TBI is lower than that for the sham-operated dataset in the cingulum (bottom row), consistent with a lower coefficient of variation and a more ordered arrangement. (This can also be contrasted with a periodic arrangement: $\Gamma_{\text{pos}} \equiv 0$ for $q < 2\pi/\bar{a}$.)

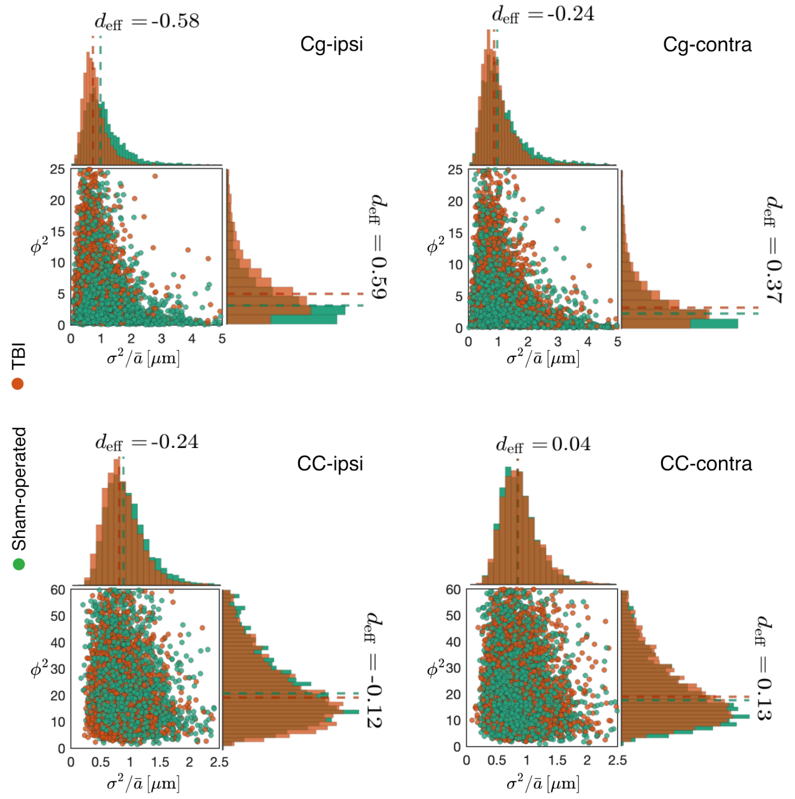

FIG. S9. **Geometric interpretation of Γ_0**. Comparisons over Cg-ipsi ($N_{\text{axon}} = 3,999$), Cg-contra ($N_{\text{axon}} = 4,580$), CC-ipsi ($N_{\text{axon}} = 2,703$), and CC-contra ($N_{\text{axon}} = 4,080$) show that TBI caused a decrease $\sigma_a^2/\bar{a}$ and decrease in the ϕ^2. Source data are provided in the Source Data file.

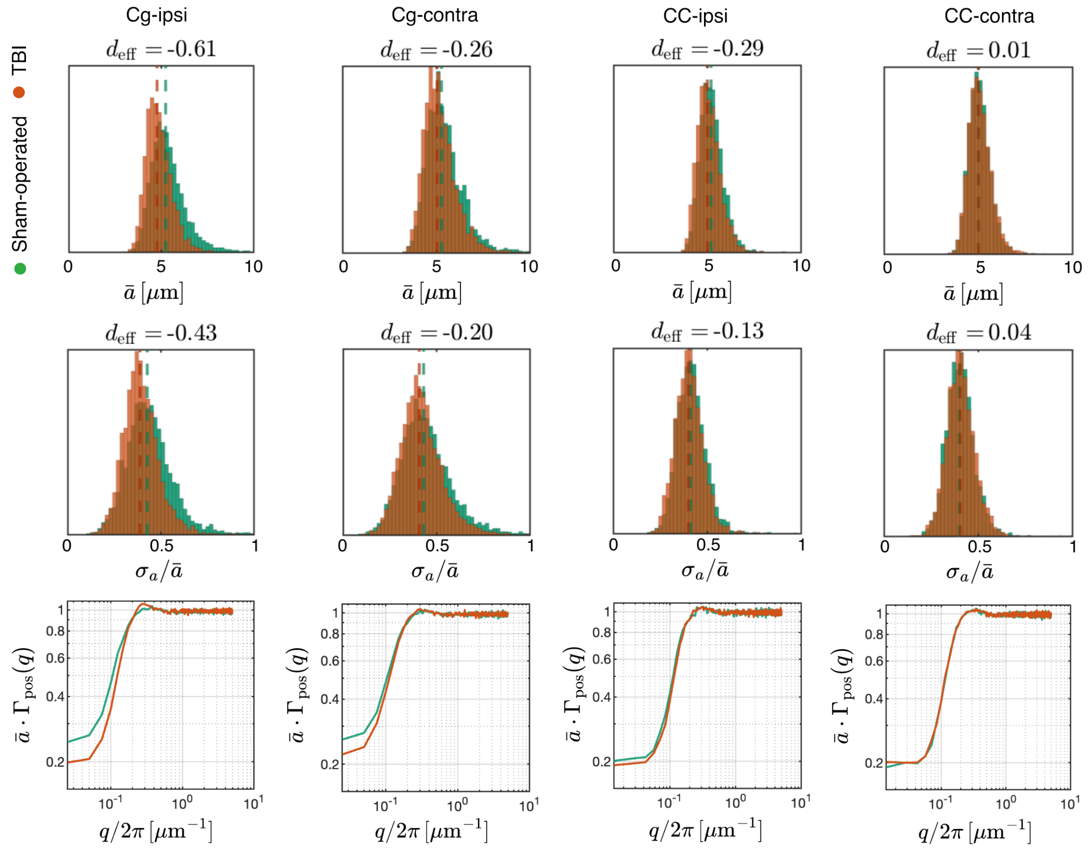

FIG. S10. **Statistics of bead positions**. The top row shows the distribution of the mean distance $\bar{a}$ between successive beads for all myelinated axons from sham-operated and TBI animals in Cg-ipsi ($N_{\text{axon}} = 3,999$), Cg-contra ($N_{\text{axon}} = 4,580$), CC-ipsi ($N_{\text{axon}} = 2,703$), and CC-contra ($N_{\text{axon}} = 4,080$) datasets; TBI caused a substantial decrease in $\bar{a}$. The middle row shows the coefficient of variation $\sigma_a/\bar{a}$ of bead positions. Interestingly, bead positions in TBI have a smaller variation than in sham-operated datasets, indicating a more ordered placement. This can also be seen from the plateau $\bar{a} \cdot \Gamma_{\text{pos}}|_{q\to 0} = (\sigma_a/\bar{a})^2$, accodring to Eq. (S13) (bottom row). The lower plateau in TBI indicates a more ordered bead placement. Source data are provided in the Source Data file.

THE HARMONIC UNDULATION MODEL

Here, we extend the treatment of Gaussian diffusion along the axonal arc-length (Appendix E of Ref. [59]) onto the case of arbitrary, time-dependent $D(t)$. To quantify the effect of axonal undulations on diffusion metrics, we introduce sinuosity ξ defined as the ratio of the arc-length $L = \int \mathrm{d}l$ of the axonal skeleton to its Euclidean length $L_x = \int \mathrm{d}x$:

$$\xi = \frac{L}{L_x} = \frac{1}{L_x} \int \sqrt{(\mathrm{d}x)^2 + |\mathrm{d}\mathbf{w}|^2}, \tag{S15}$$

where l is the coordinate along the skeleton, x is the coordinate along the main axis, and $\mathbf{w}(l)$ is the vector of the shortest distance between the skeleton and the main axis at each point l along the skeleton, Fig. S11a.

To understand the effect of undulation on diffusion along axons, we consider a sinusoidal undulation in one plane (the 1-harmonic model [59]):

$$w = w_0 \cos k_u x \tag{S16}$$

where w_0 is the undulation amplitude, $k_u = 2\pi/\lambda$, and λ is the undulation wavelength. Expanding Eq. (S15) for the 1-harmonic model, we have

$$\begin{aligned} l(x) &\simeq \int_0^x \mathrm{d}x \left(1 + \frac{(\mathrm{d}w/\mathrm{d}x)^2}{2} - \frac{(\mathrm{d}w/\mathrm{d}x)^4}{8}\right) \\ &= \int_0^x \mathrm{d}x \left(1 + 2\epsilon \sin^2 k_u x + 2\epsilon^2 \sin^4 k_u x\right) = \xi x - (\epsilon - \epsilon^2)\frac{\sin 2k_u x}{2k_u} - \epsilon^2 \frac{\sin 4k_u x}{16 k_u}, \end{aligned} \tag{S17}$$

where $\epsilon \equiv (k_u w_0/2)^2 = (\pi w_0/\lambda)^2 \ll 1$, and sinuosity $\xi \simeq 1 + \epsilon - \frac{3}{4}\epsilon^2$. We now invert $l(x)$ perturbatively up to $\mathcal{O}(\epsilon^2)$:

$$x(l) \simeq \frac{l}{\xi}\left[1 + \frac{\epsilon}{2k_u l} \sin \frac{2k_u l}{\xi} - \frac{\epsilon^2}{2k_u l}\left(\sin 2k_u l - \frac{5}{8} \sin 4k_u l\right)\right]. \tag{S18}$$

To get the above equation, we approximated $\sin 2k_u x \approx \sin(2k_u l/\xi) + (\epsilon/2)\sin 4k_u l$ up to $\mathcal{O}(\epsilon)$, and substituted $\sin 4k_u x$ with $\sin 4k_u l$ as that term is already $\mathcal{O}(\epsilon^2)$.

The cumulative axial diffusivity $D(t) \equiv \langle \delta x^2(t)\rangle/2t$ along the main axis is given in terms of

$$\langle \delta x^2(t)\rangle = \frac{1}{L}\int \left[x(l) - x(l')\right]^2 G(t, l - l')\, \mathrm{d}l\mathrm{d}l', \tag{S19}$$

where $G(t,l)$ is the disorder-averaged propagator of the Fick-Jacobs Eq. (4) taken along the arc length l (i.e. when the axonal undulations are straightened, as described in *Methods*). In other words, it is the EMT propagator (15) (in the time-space representation) of the FJ equation (11), where, instead of x, we work with the "unrolled" arc length coordinate l, and take into account scatterings off the fluctuations of $\alpha(l)$. As we stated in *Methods*, all the above analyses have been performed after such an unrolling — e.g., $\Gamma_\eta(q)$ is obtained in the arc length coordinates, with q in Fig. 2 being the Fourier variable conjugate to the cross-sectional area fluctuations along the arc length.

We now substitute $x(l)$ from Eq. (S18) into Eq. (S19). For that, we change variables to $l_+ = (l+l')/2$ and $l_- = l - l'$, and expand $[x(l) - x(l')]^2$ up to ϵ^2, setting $\xi \to 1$ whenever the term is already $\mathcal{O}(\epsilon^2)$:

$$\begin{aligned} x(l) - x(l') &\simeq \frac{l_-}{\xi} + \frac{\epsilon}{k_u \xi}\cos\frac{2k_u l_+}{\xi}\sin\frac{k_u l_-}{\xi} - \frac{\epsilon^2}{k_u}\left[\cos 2k_u l_+ \sin k_u l_- - \frac{5}{8}\cos 4k_u l_+ \sin 2k_u l_-\right] + \mathcal{O}(\epsilon^3) \quad \Rightarrow \\ [x(l) - x(l')]^2 &\simeq \left(\frac{l_-}{\xi}\right)^2 + \frac{\epsilon^2}{k_u^2}\cos^2 2k_u l_+ \sin^2 k_u l_- + \frac{2\epsilon l_-}{k_u \xi^2}\cos\frac{2k_u l_+}{\xi}\sin\frac{k_u l_-}{\xi} \\ &\quad - \frac{2\epsilon^2 l_-}{k_u}\left[\cos 2k_u l_+ \sin k_u l_- - \frac{5}{8}\cos 4k_u l_+ \sin 2k_u l_-\right] + \mathcal{O}(\epsilon^3). \end{aligned} \tag{S20}$$

Integrating Eq. (S20) with the propagator $G(t, l_-)$ in Eq. (S19) implies the averaging with respect to l_+ over large L. This cancels all the oscillating terms, such that only the first two terms survive; in the second term, $\cos^2 2k_u l_+ \to \frac{1}{2}$ after averaging, and $\sin^2 k_u l_- = \frac{1}{2}[1 - \mathrm{Re}\, e^{-2ik_u l_-}]$ yields the Fourier transform of $G(t, l_-)$. As a result,

$$\langle \delta x^2(t)\rangle \simeq \frac{1}{\xi^2}\int l_-^2 G(t, l_-)\mathrm{d}l_- + \frac{\epsilon^2}{4k_u^2}\left[1 - G(t,q)|_{q=2k_u}\right] + \mathcal{O}(\epsilon^3). \tag{S21}$$

From Eq. (S21), we conclude

$$D(t) = \frac{\langle \delta x^2(t) \rangle}{2t} \simeq \frac{1}{\xi^2} D_l(t) + \frac{\epsilon^2}{8k_u^2 t} \left[1 - G(t,q)|_{q=2k_u}\right] . \tag{S22}$$

The second term in Eq. (S22) decays at least as fast as $1/t$ for any propagator G, while the $1/\sqrt{t}$ behavior all comes from the diffusion coefficient $D(t)$ renormalized by the $1/\xi^2$ factor:

$$D(t) \simeq \frac{1}{\xi^2} \left[D_{\infty,l} + \frac{c_{D,l}}{\sqrt{t}} \right] . \tag{S23}$$

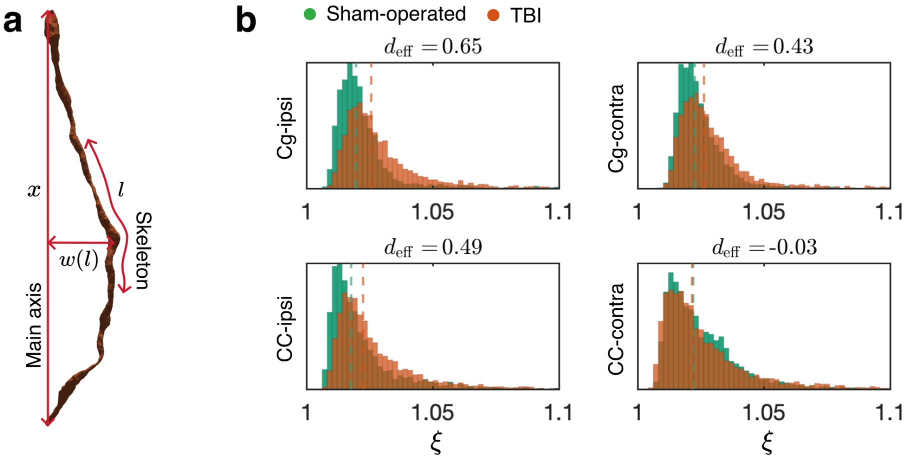

FIG. S11. **Axonal sinuosity**. TBI caused an increase in the axonal sinuosity ξ in the Cg-ipsi ($N_{\rm axon} = 3,999$), Cg-contra ($N_{\rm axon} = 4,580$), and CC-ipsi ($N_{\rm axon} = 2,703$), and had no effect on CC-contra ($N_{\rm axon} = 4,080$). Source data are provided in the Source Data file.

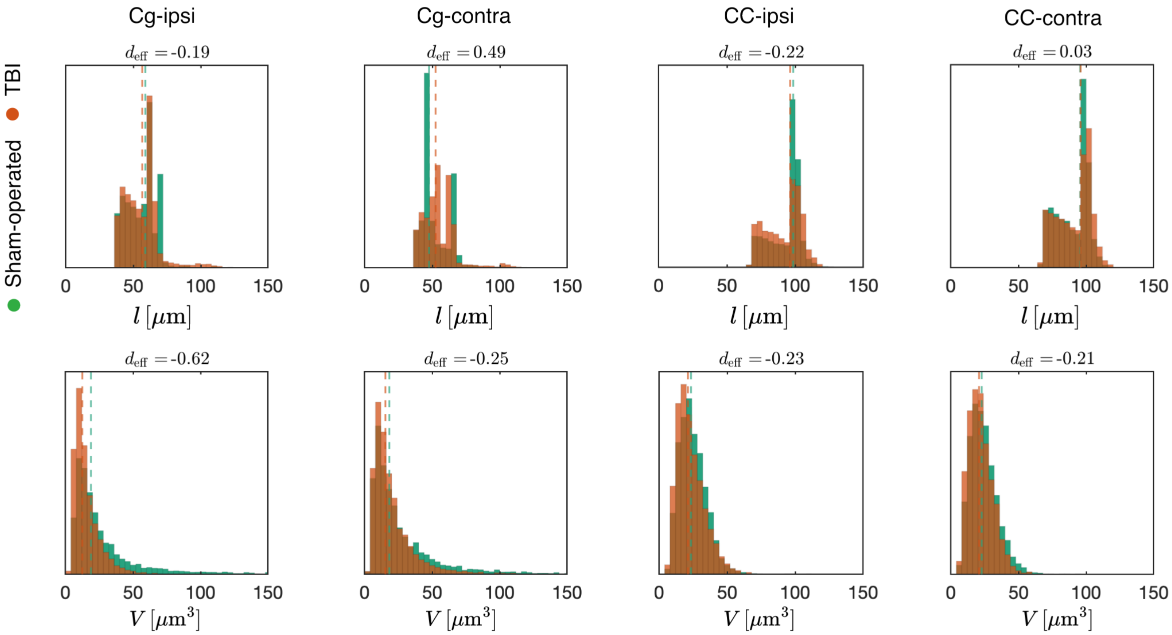

FIG. S12. **Axonal length and volume**. Distributions of axonal length l and volume V for all myelinated axons included in this study. Axons shorter than $70, \mu$m in the corpus callosum and $40, \mu$m in the cingulum were excluded. While axonal lengths remain comparable between groups, the reduced axonal volumes observed in the TBI group reflect a decrease in cross-sectional diameters. This is also shown in Fig. S8 (top row) across brain regions: Cg-ipsi ($N_{\text{axon}} = 3,999$), Cg-contra ($N_{\text{axon}} = 4,580$), CC-ipsi ($N_{\text{axon}} = 2,703$), and CC-contra ($N_{\text{axon}} = 4,080$). Source data are provided in the Source Data file.

DESCRIPTION OF DATASETS

TABLE S1. **Description of the SBEM datasets.** We collected the SBEM images from the ipsi- and contralateral corpus callosum and cingulum of two sham-operated rats and three rats with traumatic brain injury (TBI). The images from the ipsilateral hemisphere of the sham #49 rat included only the cingulum. The order of the axes is as x, y, z, where the z-axis is the EM imaging direction.

Condition	Rat ID	Tissue size (voxel^3)	Voxel size (nm)	Tissue size (µm^3)
Sham	#25 contra	$2\,044 \times 4\,096 \times 1\,306$	$50 \times 50 \times 50$	$102.2 \times 204.8 \times 65.3$
	#25 ipsi	$4\,096 \times 2\,048 \times 1\,384$	$50 \times 50 \times 50$	$204.8 \times 102.4 \times 69.2$
	#49 contra	$4\,096 \times 2\,048 \times 1\,882$	$50 \times 50 \times 50$	$204.8 \times 102.4 \times 94.1$
	#49 ipsi	$2\,048 \times 2\,048 \times 1\,210$	$50 \times 50 \times 50$	$102.4 \times 102.4 \times 60.5$
TBI	#2 contra	$4\,096 \times 2\,048 \times 1\,086$	$50 \times 50 \times 50$	$204.8 \times 102.4 \times 54.3$
	#2 ipsi	$2\,154 \times 4\,134 \times 620$	$50 \times 50 \times 50$	$107.7 \times 206.7 \times 31.0$
	#24 contra	$4\,091 \times 2\,028 \times 1\,348$	$50 \times 50 \times 50$	$204.5 \times 101.4 \times 67.4$
	#24 ipsi	$2\,946 \times 2\,162 \times 1\,250$	$50 \times 50 \times 50$	$147.3 \times 108.1 \times 62.5$
	#28 contra	$4\,096 \times 2\,048 \times 1\,278$	$50 \times 50 \times 50$	$204.8 \times 102.4 \times 63.9$
	#28 ipsi	$4\,075 \times 2\,000 \times 1\,300$	$50 \times 50 \times 50$	$203.7 \times 100.0 \times 65.0$
